# Tetra­kis[μ-2-(3,4-dimeth­oxy­phen­yl)acetato]-κ^3^ *O* ^1^,*O* ^1′^:*O* ^1^;κ^3^ *O* ^1^:*O* ^1^,*O* ^1′^;κ^4^ *O* ^1^:*O* ^1′^-bis­{[2-(3,4-dimeth­oxy­phen­yl)acetato-κ^2^ *O* ^1^,*O* ^1′^](1,10-phenanthroline-κ^2^ *N*,*N*′)erbium(III)}

**DOI:** 10.1107/S1600536810040298

**Published:** 2010-10-13

**Authors:** Jia-Lu Liu, Hua-Qiong Li, Guo-Liang Zhao

**Affiliations:** a College of Chemistry and Life Sciences, Zhejiang Normal University Xingzhi College, Zhejiang Normal University, Jinhua 321004, People’s Republic of China

## Abstract

In the dimeric centrosymmetric title complex, [Er_2_(C_10_H_11_O_4_)_6_(C_12_H_8_N_2_)_2_], the Er^III^ ion is nine-coordinated by five 2-(3,4-dimeth­oxy­lphen­yl)acetic acid (DMPA) ligands *via* seven O atoms and two N atoms from a bis-chelating 1,10-phenanthroline (phen) ligand in a distorted tricapped trigonal-prismatic geometry. The DMPA ligands are coordinated to the Er^III^ ion in bis-chelate, bridging and bridging tridentate modes. Relatively weak intra­molecular C—H⋯O inter­actions reinforce the stability of the mol­ecular structure. Inter­molecular C—H⋯O inter­actions are also observed.

## Related literature

For the properties of carb­oxy­lic metal–organic complexes, see: Aoki *et al.* (2004 ▶); Yao *et al.* (2008 ▶). For related structures, see: Xia *et al.* (2008 ▶); Liu *et al.* (2007 ▶); Li *et al.* (2008 ▶).
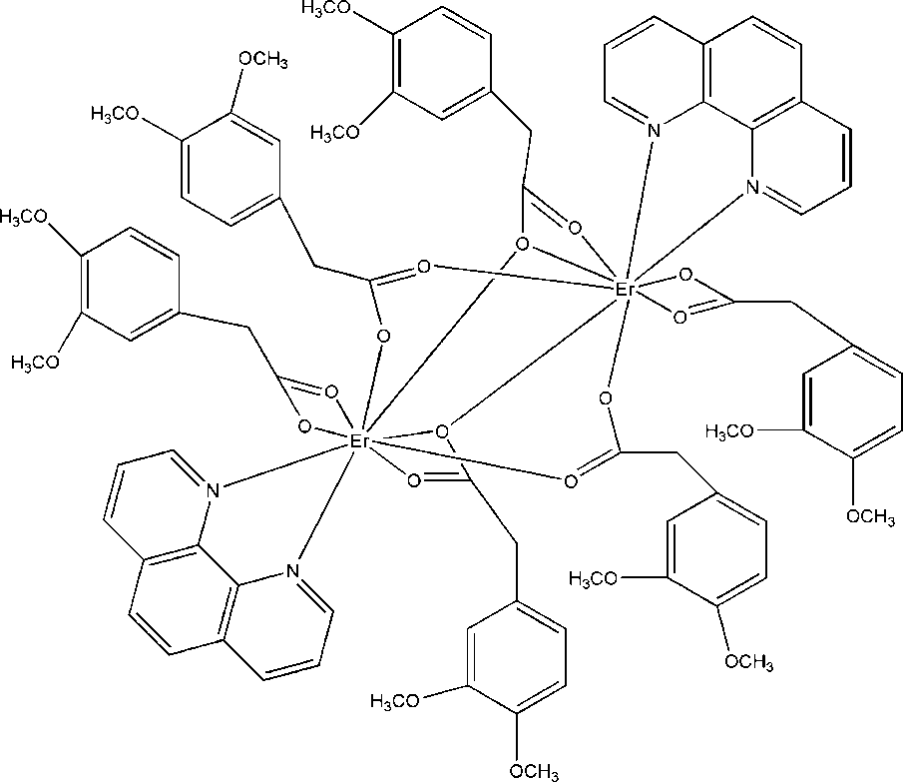


## Experimental

### 

#### Crystal data

[Er_2_(C_10_H_11_O_4_)_6_(C_12_H_8_N_2_)_2_]
*M* *_r_* = 1866.06
Triclinic, 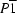

*a* = 12.3101 (1) Å
*b* = 12.3646 (1) Å
*c* = 14.6260 (2) Å
α = 91.233 (1)°
β = 103.469 (1)°
γ = 114.876 (1)°
*V* = 1946.02 (4) Å^3^
*Z* = 1
Mo *K*α radiation
μ = 2.22 mm^−1^
*T* = 296 K
0.60 × 0.23 × 0.17 mm

#### Data collection

Bruker APEXII area-detector diffractometer
Absorption correction: multi-scan (*SADABS*; Sheldrick, 1996 ▶) *T*_min_ = 0.547, *T*_max_ = 0.681
31446 measured reflections
9031 independent reflections
7784 reflections with *I* > 2σ(*I*)
*R*_int_ = 0.026

#### Refinement

*R*[*F* ^2^ > 2σ(*F* ^2^)] = 0.023
*wR*(*F* ^2^) = 0.059
*S* = 1.00
9031 reflections
514 parameters
H-atom parameters constrained
Δρ_max_ = 0.86 e Å^−3^
Δρ_min_ = −0.46 e Å^−3^

### 

Data collection: *APEX2* (Bruker, 2006 ▶); cell refinement: *SAINT* (Bruker, 2006 ▶); data reduction: *SAINT*; program(s) used to solve structure: *SHELXS97* (Sheldrick, 2008 ▶); program(s) used to refine structure: *SHELXL97* (Sheldrick, 2008 ▶); molecular graphics: *SHELXTL* (Sheldrick, 2008 ▶); software used to prepare material for publication: *SHELXL97*.

## Supplementary Material

Crystal structure: contains datablocks I, global. DOI: 10.1107/S1600536810040298/pv2331sup1.cif

Structure factors: contains datablocks I. DOI: 10.1107/S1600536810040298/pv2331Isup2.hkl

Additional supplementary materials:  crystallographic information; 3D view; checkCIF report

## Figures and Tables

**Table 1:** Hydrogen-bond geometry (Å, °)

*D*—H⋯*A*	*D*—H	H⋯*A*	*D*⋯*A*	*D*—H⋯*A*
C1—H1*A*⋯O6^i^	0.96	2.54	3.312 (4)	137
C16—H16*A*⋯O3^ii^	0.93	2.52	3.421 (3)	163
C18—H18*C*⋯O3^ii^	0.96	2.38	3.267 (4)	153
C28—H28*A*⋯O1^iii^	0.96	2.48	3.353 (6)	151
C28—H28*A*⋯O2^iii^	0.96	2.44	3.220 (5)	138
C33—H33*A*⋯O7^iv^	0.93	2.37	3.218 (3)	152
C40—H40*A*⋯O11^v^	0.93	2.36	2.998 (4)	125

Symmetry codes: (i) 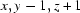; (ii) 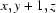; (iii) 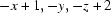; (iv) 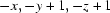; (v) 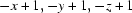.

**Fig. 1.**
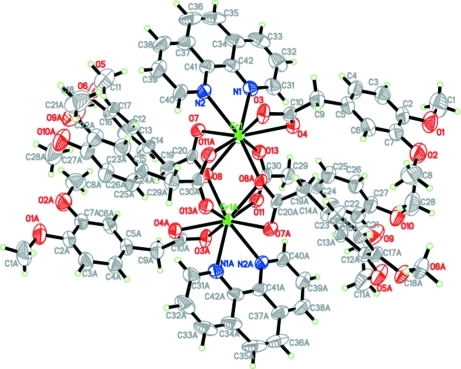
The molecular structure of the title complex, showing the atom-labelling scheme. Displacement ellipsoids are drawn at the 50% probability level.

## supplementary crystallographic information

### Comment

Interest in the design and synthesis of carboxylic metal-organic complexes has been increasing for decades owing to their potential practical applications, such as fluorescence (Aoki *et al.*, 2004), magnetism (Yao *et al.*, 2008). In this paper, we report the preparation and crystal structure of a new erbium^III^ complex with the ligands 3,4-dimethoxylphenyl acetic acid (DMPA) (homoveratric acid) and 1,10-phenanthroline (phen). The crystal structures of several rare earth elements closely related to the title compound have been reported recently (Xia *et al.*, 2008; Liu *et al.*, 2007).

The title compound is a dimeric complex which consists of two central erbium^III^ atoms coordinated with six 3,4-dimethoxylphenyl acetate ions and two phen molecules. The DMPA ligands are coordinated by three modes, bis-chelate, bridging and bridging tridentate. Each erbium atom is nine coordinated, seven O atoms from five DMPA molecules and two N atoms from a phen molecule (Fig. 1). The erbium^III^ atom is in a distorted capped square prism environment. The Er—O bond lengths lie in the range 2.3006 (15)– 2.5020 (18) Å while the Er—N distances lie in the range 2.5135 (19)–2.588 (2) Å. There are no classical hydrogen bonds in the crystal structure. The most significant intermolecular interactions are C—H···O hydrogen bonds which lend stability to the structure (Table 1).

### Experimental

All reagents and solvents used were of commercially available quality and wre not purified before using. The title compound was obtained by adding Er_2_O_3_ (0.5 mmol), homoveratric acid (3.0 mmol) and 1,10-phenanthroline (1.0 mmol) dissolved in 30 ml water, sealed in a 50 ml stainless steel reactor and kept at 433 K for three days. Then the reactor was cooled to room temperature at a speed of 5° per hour. The solution was filtered and the deposition was washed with ethanol to obtain pink block crystals of the title compound.

### Refinement

The H atoms were positioned geometrically and refined using a riding model with C—H = 0.97, 0.96 and 0.93 Å for methylene, methyl and aryl H-atoms, respectively and (*U*_iso_(H) = 1.5*U*_eq_(methyl C) or 1.2*U*_eq_(methylene/aryl C).

### Crystal data

**Table:**

[Er_2_(C_10_H_11_O_4_)_6_(C_12_H_8_N_2_)_2_]	*Z* = 1
*M**_r_* = 1866.06	*F*(000) = 942
Triclinic, *P*1	*D*_x_ = 1.592 Mg m^−^^3^
Hall symbol: -P 1	Mo *K*α radiation, λ = 0.71073 Å
*a* = 12.3101 (1) Å	Cell parameters from 9977 reflections
*b* = 12.3646 (1) Å	θ = 1.8–27.6°
*c* = 14.6260 (2) Å	µ = 2.22 mm^−^^1^
α = 91.233 (1)°	*T* = 296 K
β = 103.469 (1)°	Block, pink
γ = 114.876 (1)°	0.60 × 0.23 × 0.17 mm
*V* = 1946.02 (4) Å^3^	

### Data collection

**Table:**

Bruker APEXII area-detector diffractometer	9031 independent reflections
Radiation source: fine-focus sealed tube	7784 reflections with *I* > 2σ(*I*)
graphite	*R*_int_ = 0.026
φ and ω scans	θ_max_ = 27.6°, θ_min_ = 1.8°
Absorption correction: multi-scan (*SADABS*; Sheldrick, 1996)	*h* = −16→16
*T*_min_ = 0.547, *T*_max_ = 0.681	*k* = −16→16
31446 measured reflections	*l* = −19→18

### Refinement

**Table:**

Refinement on *F*^2^	Primary atom site location: structure-invariant direct methods
Least-squares matrix: full	Secondary atom site location: difference Fourier map
*R*[*F*^2^ > 2σ(*F*^2^)] = 0.023	Hydrogen site location: inferred from neighbouring sites
*wR*(*F*^2^) = 0.059	H-atom parameters constrained
*S* = 1.00	*w* = 1/[σ^2^(*F*_o_^2^) + (0.0319*P*)^2^ + 0.6854*P*] where *P* = (*F*_o_^2^ + 2*F*_c_^2^)/3
9031 reflections	(Δ/σ)_max_ = 0.001
514 parameters	Δρ_max_ = 0.86 e Å^−^^3^
0 restraints	Δρ_min_ = −0.46 e Å^−^^3^

### Special details

**Table:**

Geometry. All e.s.d.'s (except the e.s.d. in the dihedral angle between two l.s. planes) are estimated using the full covariance matrix. The cell e.s.d.'s are taken into account individually in the estimation of e.s.d.'s in distances, angles and torsion angles; correlations between e.s.d.'s in cell parameters are only used when they are defined by crystal symmetry. An approximate (isotropic) treatment of cell e.s.d.'s is used for estimating e.s.d.'s involving l.s. planes.
Refinement. Refinement of *F*^2^ against ALL reflections. The weighted *R*-factor *wR* and goodness of fit *S* are based on *F*^2^, conventional *R*-factors *R* are based on *F*, with *F* set to zero for negative *F*^2^. The threshold expression of *F*^2^ > σ(*F*^2^) is used only for calculating *R*-factors(gt) *etc*. and is not relevant to the choice of reflections for refinement. *R*-factors based on *F*^2^ are statistically about twice as large as those based on *F*, and *R*- factors based on ALL data will be even larger.

### Fractional atomic coordinates and isotropic or equivalent isotropic displacement parameters (Å^2^)

**Table:**

	*x*	*y*	*z*	*U*_iso_*/*U*_eq_	
Er1	0.323792 (9)	0.396219 (10)	0.468848 (7)	0.03886 (4)	
C20	0.4065 (2)	0.6372 (2)	0.42582 (16)	0.0393 (5)	
O13	0.38701 (15)	0.51937 (17)	0.61236 (12)	0.0457 (4)	
O8	0.48695 (15)	0.59812 (16)	0.45693 (12)	0.0446 (4)	
C10	0.2382 (2)	0.1683 (2)	0.52142 (18)	0.0428 (5)	
O6	0.13086 (17)	0.94341 (16)	0.19422 (13)	0.0499 (4)	
O7	0.29483 (15)	0.57170 (16)	0.41590 (13)	0.0478 (4)	
O4	0.27956 (16)	0.25477 (16)	0.58501 (12)	0.0459 (4)	
O5	0.1108 (2)	0.73691 (18)	0.13200 (15)	0.0657 (6)	
C30	0.4893 (2)	0.5821 (2)	0.67136 (17)	0.0434 (6)	
N2	0.14185 (18)	0.34244 (19)	0.31818 (16)	0.0452 (5)	
O3	0.23958 (19)	0.18490 (18)	0.43671 (12)	0.0537 (5)	
C19	0.4540 (2)	0.7642 (2)	0.4028 (2)	0.0539 (7)	
H19A	0.5025	0.8178	0.4617	0.065*	
H19B	0.5104	0.7736	0.3637	0.065*	
O1	0.2656 (2)	−0.0141 (3)	0.93214 (16)	0.0803 (7)	
N1	0.11826 (19)	0.3593 (2)	0.49755 (16)	0.0485 (5)	
C31	0.1045 (3)	0.3605 (3)	0.5845 (2)	0.0598 (7)	
H31A	0.1704	0.3682	0.6349	0.072*	
C41	0.0342 (2)	0.3398 (2)	0.3297 (2)	0.0440 (6)	
C17	0.2058 (2)	0.9018 (2)	0.25119 (17)	0.0391 (5)	
C16	0.2857 (2)	0.9600 (2)	0.33756 (19)	0.0495 (6)	
H16A	0.2900	1.0320	0.3624	0.059*	
C12	0.1976 (2)	0.7912 (2)	0.21669 (17)	0.0417 (5)	
C24	0.5329 (2)	0.5294 (3)	0.83566 (18)	0.0482 (6)	
C14	0.3603 (2)	0.8066 (2)	0.35290 (19)	0.0443 (6)	
C5	0.2042 (3)	0.0264 (2)	0.64677 (19)	0.0511 (6)	
O9	0.7502 (3)	0.5462 (3)	1.0651 (2)	0.0952 (9)	
C13	0.2752 (2)	0.7464 (2)	0.26708 (18)	0.0436 (5)	
H13A	0.2704	0.6738	0.2428	0.052*	
O2	0.3979 (2)	−0.0555 (2)	0.83738 (18)	0.0773 (6)	
C7	0.3136 (3)	−0.0197 (3)	0.7876 (2)	0.0548 (7)	
C15	0.3609 (3)	0.9109 (3)	0.3883 (2)	0.0558 (7)	
H15A	0.4129	0.9499	0.4479	0.067*	
C40	0.1520 (3)	0.3342 (2)	0.2306 (2)	0.0515 (6)	
H40A	0.2259	0.3375	0.2221	0.062*	
C37	−0.0644 (2)	0.3288 (2)	0.2518 (2)	0.0549 (7)	
O10	0.6576 (2)	0.3197 (2)	1.00775 (17)	0.0818 (7)	
C18	0.1621 (3)	1.0676 (3)	0.2155 (2)	0.0599 (7)	
H18A	0.1049	1.0880	0.1713	0.090*	
H18B	0.2450	1.1155	0.2109	0.090*	
H18C	0.1575	1.0831	0.2788	0.090*	
C28	0.6061 (4)	0.1936 (4)	0.9784 (3)	0.0991 (14)	
H28A	0.6444	0.1579	1.0251	0.149*	
H28B	0.6211	0.1799	0.9187	0.149*	
H28C	0.5183	0.1581	0.9718	0.149*	
C22	0.6623 (3)	0.5063 (3)	0.9784 (2)	0.0626 (8)	
C29	0.4884 (3)	0.6070 (3)	0.77314 (19)	0.0533 (7)	
H29A	0.4048	0.5906	0.7752	0.064*	
H29B	0.5420	0.6912	0.7971	0.064*	
C9	0.1860 (3)	0.0392 (3)	0.5431 (2)	0.0598 (7)	
H9A	0.0977	−0.0002	0.5119	0.072*	
H9B	0.2249	−0.0026	0.5160	0.072*	
C42	0.0212 (2)	0.3471 (2)	0.4237 (2)	0.0470 (6)	
C6	0.2942 (3)	−0.0074 (2)	0.6926 (2)	0.0538 (7)	
H6A	0.3428	−0.0221	0.6586	0.065*	
C23	0.6219 (3)	0.5780 (3)	0.9218 (2)	0.0564 (7)	
H23A	0.6551	0.6600	0.9421	0.068*	
C39	0.0579 (3)	0.3208 (3)	0.1499 (2)	0.0618 (7)	
H39A	0.0685	0.3131	0.0895	0.074*	
C27	0.6132 (3)	0.3837 (3)	0.9484 (2)	0.0598 (7)	
C2	0.2414 (3)	0.0033 (3)	0.8388 (2)	0.0550 (7)	
C8	0.4842 (3)	−0.0659 (4)	0.7925 (3)	0.0870 (11)	
H8A	0.5377	−0.0919	0.8352	0.131*	
H8B	0.5332	0.0108	0.7757	0.131*	
H8C	0.4402	−0.1237	0.7363	0.131*	
C25	0.4838 (3)	0.4076 (3)	0.8068 (2)	0.0585 (7)	
H25A	0.4237	0.3738	0.7493	0.070*	
C3	0.1533 (3)	0.0392 (3)	0.7943 (2)	0.0606 (7)	
H3A	0.1059	0.0558	0.8285	0.073*	
C38	−0.0498 (3)	0.3191 (3)	0.1611 (2)	0.0635 (8)	
H38A	−0.1130	0.3116	0.1084	0.076*	
C26	0.5234 (3)	0.3349 (3)	0.8629 (2)	0.0641 (8)	
H26A	0.4892	0.2527	0.8428	0.077*	
C33	−0.1011 (3)	0.3428 (3)	0.5310 (3)	0.0731 (10)	
H33A	−0.1738	0.3383	0.5429	0.088*	
C36	−0.1735 (3)	0.3303 (3)	0.2695 (3)	0.0725 (10)	
H36A	−0.2368	0.3273	0.2187	0.087*	
C4	0.1346 (3)	0.0507 (3)	0.6987 (2)	0.0604 (7)	
H4A	0.0748	0.0751	0.6691	0.073*	
C35	−0.1856 (3)	0.3360 (3)	0.3573 (3)	0.0763 (11)	
H35A	−0.2576	0.3367	0.3665	0.092*	
C34	−0.0903 (2)	0.3413 (2)	0.4383 (3)	0.0599 (8)	
C21	0.7933 (4)	0.6639 (4)	1.1050 (3)	0.1027 (14)	
H21A	0.8541	0.6794	1.1644	0.154*	
H21B	0.7252	0.6767	1.1156	0.154*	
H21C	0.8303	0.7174	1.0628	0.154*	
C32	−0.0054 (3)	0.3508 (3)	0.6039 (3)	0.0713 (9)	
H32A	−0.0127	0.3498	0.6657	0.086*	
C11	0.0805 (4)	0.6155 (3)	0.1040 (3)	0.0807 (11)	
H11A	0.0201	0.5878	0.0434	0.121*	
H11B	0.0468	0.5674	0.1502	0.121*	
H11C	0.1540	0.6088	0.0994	0.121*	
C1	0.1893 (4)	−0.0026 (4)	0.9857 (3)	0.0985 (13)	
H1A	0.2161	−0.0168	1.0495	0.148*	
H1B	0.1048	−0.0602	0.9578	0.148*	
H1C	0.1947	0.0772	0.9866	0.148*	
O11	0.59453 (15)	0.62444 (18)	0.65483 (12)	0.0506 (4)	

### Atomic displacement parameters (Å^2^)

**Table:**

	*U*^11^	*U*^22^	*U*^33^	*U*^12^	*U*^13^	*U*^23^
Er1	0.04056 (6)	0.05999 (8)	0.03747 (7)	0.03744 (5)	0.01798 (4)	0.02142 (5)
C20	0.0433 (12)	0.0584 (15)	0.0339 (12)	0.0370 (11)	0.0129 (9)	0.0172 (11)
O13	0.0462 (9)	0.0663 (11)	0.0440 (10)	0.0377 (9)	0.0209 (8)	0.0172 (8)
O8	0.0462 (9)	0.0686 (11)	0.0445 (9)	0.0443 (9)	0.0194 (7)	0.0268 (8)
C10	0.0418 (12)	0.0574 (16)	0.0415 (14)	0.0314 (11)	0.0134 (10)	0.0163 (12)
O6	0.0578 (10)	0.0528 (11)	0.0477 (10)	0.0380 (9)	0.0026 (8)	0.0126 (8)
O7	0.0417 (9)	0.0594 (11)	0.0595 (11)	0.0357 (8)	0.0169 (8)	0.0236 (9)
O4	0.0544 (10)	0.0561 (11)	0.0413 (10)	0.0338 (8)	0.0191 (8)	0.0156 (9)
O5	0.0816 (14)	0.0508 (12)	0.0545 (12)	0.0366 (11)	−0.0147 (10)	−0.0007 (9)
C30	0.0524 (14)	0.0620 (16)	0.0395 (13)	0.0423 (13)	0.0207 (11)	0.0212 (12)
N2	0.0429 (10)	0.0523 (12)	0.0533 (13)	0.0318 (10)	0.0141 (9)	0.0193 (10)
O3	0.0765 (12)	0.0665 (12)	0.0352 (10)	0.0444 (10)	0.0195 (9)	0.0196 (9)
C19	0.0426 (13)	0.0563 (16)	0.0697 (19)	0.0326 (12)	0.0052 (12)	0.0174 (14)
O1	0.0925 (16)	0.115 (2)	0.0494 (13)	0.0544 (15)	0.0284 (11)	0.0366 (13)
N1	0.0455 (11)	0.0597 (13)	0.0610 (14)	0.0348 (10)	0.0279 (10)	0.0220 (11)
C31	0.0591 (16)	0.075 (2)	0.071 (2)	0.0411 (15)	0.0392 (15)	0.0243 (16)
C41	0.0361 (11)	0.0365 (12)	0.0649 (17)	0.0228 (10)	0.0096 (11)	0.0134 (11)
C17	0.0422 (11)	0.0470 (13)	0.0396 (13)	0.0293 (10)	0.0119 (10)	0.0158 (10)
C16	0.0559 (14)	0.0527 (15)	0.0485 (15)	0.0365 (13)	0.0048 (11)	0.0030 (12)
C12	0.0458 (12)	0.0441 (13)	0.0402 (13)	0.0249 (11)	0.0099 (10)	0.0118 (11)
C24	0.0515 (13)	0.0699 (18)	0.0362 (13)	0.0335 (13)	0.0210 (11)	0.0172 (12)
C14	0.0404 (12)	0.0483 (14)	0.0510 (15)	0.0272 (11)	0.0084 (10)	0.0180 (12)
C5	0.0611 (15)	0.0474 (15)	0.0462 (15)	0.0217 (12)	0.0202 (12)	0.0161 (12)
O9	0.0987 (19)	0.0903 (19)	0.0764 (17)	0.0460 (16)	−0.0207 (14)	−0.0072 (15)
C13	0.0495 (13)	0.0421 (13)	0.0488 (14)	0.0284 (11)	0.0139 (11)	0.0148 (11)
O2	0.0752 (14)	0.0984 (17)	0.0823 (16)	0.0546 (13)	0.0297 (12)	0.0420 (14)
C7	0.0564 (15)	0.0543 (16)	0.0589 (17)	0.0259 (13)	0.0202 (13)	0.0225 (13)
C15	0.0576 (15)	0.0608 (17)	0.0488 (16)	0.0372 (14)	−0.0076 (12)	0.0003 (13)
C40	0.0538 (14)	0.0599 (17)	0.0509 (16)	0.0355 (13)	0.0106 (12)	0.0177 (13)
C37	0.0392 (12)	0.0402 (14)	0.080 (2)	0.0204 (11)	0.0007 (13)	0.0076 (13)
O10	0.0985 (18)	0.0833 (17)	0.0658 (15)	0.0521 (14)	0.0009 (12)	0.0231 (12)
C18	0.0777 (19)	0.0581 (17)	0.0593 (18)	0.0479 (16)	0.0101 (14)	0.0155 (14)
C28	0.119 (3)	0.083 (3)	0.095 (3)	0.050 (3)	0.012 (3)	0.040 (2)
C22	0.0589 (16)	0.079 (2)	0.0508 (17)	0.0343 (16)	0.0077 (13)	0.0156 (15)
C29	0.0659 (16)	0.0726 (18)	0.0462 (15)	0.0471 (15)	0.0265 (13)	0.0179 (13)
C9	0.0725 (18)	0.0563 (17)	0.0470 (16)	0.0248 (14)	0.0158 (14)	0.0124 (13)
C42	0.0362 (11)	0.0379 (13)	0.0766 (19)	0.0222 (10)	0.0205 (12)	0.0140 (12)
C6	0.0624 (16)	0.0504 (15)	0.0588 (17)	0.0268 (13)	0.0293 (13)	0.0198 (13)
C23	0.0593 (16)	0.0619 (18)	0.0491 (16)	0.0279 (14)	0.0140 (13)	0.0061 (13)
C39	0.0675 (18)	0.0643 (19)	0.0539 (17)	0.0357 (15)	0.0030 (14)	0.0139 (14)
C27	0.0652 (17)	0.074 (2)	0.0461 (16)	0.0363 (16)	0.0123 (13)	0.0196 (14)
C2	0.0620 (16)	0.0591 (17)	0.0479 (16)	0.0260 (14)	0.0217 (13)	0.0218 (13)
C8	0.070 (2)	0.096 (3)	0.115 (3)	0.051 (2)	0.030 (2)	0.028 (2)
C25	0.0655 (17)	0.0656 (19)	0.0435 (15)	0.0305 (15)	0.0091 (13)	0.0111 (14)
C3	0.0681 (18)	0.074 (2)	0.0573 (18)	0.0394 (16)	0.0315 (14)	0.0205 (15)
C38	0.0523 (15)	0.0535 (17)	0.074 (2)	0.0259 (13)	−0.0080 (14)	0.0107 (15)
C26	0.077 (2)	0.0621 (19)	0.0504 (17)	0.0314 (16)	0.0094 (14)	0.0122 (14)
C33	0.0485 (16)	0.0600 (19)	0.129 (3)	0.0275 (14)	0.0486 (19)	0.0112 (19)
C36	0.0358 (13)	0.0618 (19)	0.112 (3)	0.0264 (13)	−0.0030 (16)	−0.0045 (19)
C4	0.0664 (17)	0.073 (2)	0.0565 (18)	0.0415 (16)	0.0197 (14)	0.0242 (15)
C35	0.0327 (13)	0.0607 (19)	0.135 (4)	0.0248 (13)	0.0141 (17)	−0.003 (2)
C34	0.0391 (13)	0.0462 (15)	0.104 (3)	0.0224 (11)	0.0286 (15)	0.0094 (15)
C21	0.090 (3)	0.121 (4)	0.071 (3)	0.040 (3)	−0.010 (2)	−0.024 (2)
C32	0.073 (2)	0.075 (2)	0.094 (3)	0.0404 (17)	0.057 (2)	0.0218 (18)
C11	0.096 (3)	0.0539 (19)	0.077 (2)	0.0381 (18)	−0.0111 (19)	−0.0072 (17)
C1	0.134 (4)	0.129 (4)	0.055 (2)	0.066 (3)	0.046 (2)	0.028 (2)
O11	0.0455 (9)	0.0815 (13)	0.0406 (10)	0.0387 (9)	0.0181 (7)	0.0174 (9)

### Geometric parameters (Å, °)

**Table:**

Er1—O8^i^	2.3006 (15)	C7—C6	1.376 (4)
Er1—O11^i^	2.3220 (16)	C7—C2	1.395 (4)
Er1—O13	2.3313 (18)	C15—H15A	0.9300
Er1—O3	2.359 (2)	C40—C39	1.399 (4)
Er1—O4	2.4494 (17)	C40—H40A	0.9300
Er1—O7	2.4554 (16)	C37—C38	1.388 (5)
Er1—O8	2.5020 (18)	C37—C36	1.432 (4)
Er1—N1	2.5135 (19)	O10—C27	1.361 (4)
Er1—N2	2.588 (2)	O10—C28	1.425 (5)
Er1—C10	2.760 (3)	C18—H18A	0.9600
Er1—C20	2.853 (2)	C18—H18B	0.9600
Er1—Er1^i^	3.8637 (2)	C18—H18C	0.9600
C20—O7	1.238 (3)	C28—H28A	0.9600
C20—O8	1.275 (3)	C28—H28B	0.9600
C20—C19	1.504 (4)	C28—H28C	0.9600
O13—C30	1.255 (3)	C22—C23	1.389 (4)
O8—Er1^i^	2.3006 (15)	C22—C27	1.390 (5)
C10—O4	1.243 (3)	C29—H29A	0.9700
C10—O3	1.263 (3)	C29—H29B	0.9700
C10—C9	1.521 (4)	C9—H9A	0.9700
O6—C17	1.367 (3)	C9—H9B	0.9700
O6—C18	1.425 (3)	C42—C34	1.409 (3)
O5—C12	1.368 (3)	C6—H6A	0.9300
O5—C11	1.411 (4)	C23—H23A	0.9300
C30—O11	1.261 (3)	C39—C38	1.366 (4)
C30—C29	1.517 (3)	C39—H39A	0.9300
N2—C40	1.322 (3)	C27—C26	1.382 (4)
N2—C41	1.362 (3)	C2—C3	1.374 (4)
C19—C14	1.507 (3)	C8—H8A	0.9600
C19—H19A	0.9700	C8—H8B	0.9600
C19—H19B	0.9700	C8—H8C	0.9600
O1—C2	1.370 (3)	C25—C26	1.391 (4)
O1—C1	1.404 (4)	C25—H25A	0.9300
N1—C31	1.322 (4)	C3—C4	1.384 (4)
N1—C42	1.363 (3)	C3—H3A	0.9300
C31—C32	1.402 (4)	C38—H38A	0.9300
C31—H31A	0.9300	C26—H26A	0.9300
C41—C37	1.414 (4)	C33—C32	1.359 (5)
C41—C42	1.427 (4)	C33—C34	1.394 (5)
C17—C16	1.367 (4)	C33—H33A	0.9300
C17—C12	1.402 (3)	C36—C35	1.331 (5)
C16—C15	1.395 (3)	C36—H36A	0.9300
C16—H16A	0.9300	C4—H4A	0.9300
C12—C13	1.378 (3)	C35—C34	1.439 (5)
C24—C25	1.378 (4)	C35—H35A	0.9300
C24—C23	1.386 (4)	C21—H21A	0.9600
C24—C29	1.518 (4)	C21—H21B	0.9600
C14—C13	1.385 (4)	C21—H21C	0.9600
C14—C15	1.376 (4)	C32—H32A	0.9300
C5—C6	1.381 (4)	C11—H11A	0.9600
C5—C4	1.382 (4)	C11—H11B	0.9600
C5—C9	1.502 (4)	C11—H11C	0.9600
O9—C22	1.381 (4)	C1—H1A	0.9600
O9—C21	1.382 (5)	C1—H1B	0.9600
C13—H13A	0.9300	C1—H1C	0.9600
O2—C7	1.360 (3)	O11—Er1^i^	2.3220 (16)
O2—C8	1.418 (4)		
			
O8^i^—Er1—O11^i^	75.72 (6)	C15—C14—C19	121.1 (2)
O8^i^—Er1—O13	75.88 (6)	C6—C5—C4	118.7 (3)
O11^i^—Er1—O13	138.54 (6)	C6—C5—C9	119.9 (3)
O8^i^—Er1—O3	89.35 (6)	C4—C5—C9	121.4 (3)
O11^i^—Er1—O3	79.07 (7)	C22—O9—C21	118.5 (3)
O13—Er1—O3	129.90 (6)	C12—C13—C14	121.6 (2)
O8^i^—Er1—O4	75.22 (6)	C12—C13—H13A	119.2
O11^i^—Er1—O4	123.98 (6)	C14—C13—H13A	119.2
O13—Er1—O4	75.99 (6)	C7—O2—C8	118.1 (3)
O3—Er1—O4	53.92 (6)	O2—C7—C6	125.2 (3)
O8^i^—Er1—O7	124.19 (6)	O2—C7—C2	115.6 (3)
O11^i^—Er1—O7	93.59 (6)	C6—C7—C2	119.2 (3)
O13—Er1—O7	78.30 (6)	C14—C15—C16	122.0 (2)
O3—Er1—O7	143.05 (6)	C14—C15—H15A	119.0
O4—Er1—O7	142.12 (6)	C16—C15—H15A	119.0
O8^i^—Er1—O8	72.95 (6)	N2—C40—C39	123.6 (3)
O11^i^—Er1—O8	71.53 (6)	N2—C40—H40A	118.2
O13—Er1—O8	71.64 (6)	C39—C40—H40A	118.2
O3—Er1—O8	148.53 (6)	C38—C37—C41	118.1 (3)
O4—Er1—O8	138.95 (6)	C38—C37—C36	122.9 (3)
O7—Er1—O8	52.09 (5)	C41—C37—C36	119.0 (3)
O8^i^—Er1—N1	141.53 (7)	C27—O10—C28	116.6 (3)
O11^i^—Er1—N1	139.61 (7)	O6—C18—H18A	109.5
O13—Er1—N1	78.63 (7)	O6—C18—H18B	109.5
O3—Er1—N1	85.34 (7)	H18A—C18—H18B	109.5
O4—Er1—N1	70.95 (6)	O6—C18—H18C	109.5
O7—Er1—N1	77.09 (6)	H18A—C18—H18C	109.5
O8—Er1—N1	124.64 (6)	H18B—C18—H18C	109.5
O8^i^—Er1—N2	150.03 (6)	O10—C28—H28A	109.5
O11^i^—Er1—N2	75.75 (6)	O10—C28—H28B	109.5
O13—Er1—N2	133.22 (6)	H28A—C28—H28B	109.5
O3—Er1—N2	76.40 (6)	O10—C28—H28C	109.5
O4—Er1—N2	114.34 (6)	H28A—C28—H28C	109.5
O7—Er1—N2	66.72 (6)	H28B—C28—H28C	109.5
O8—Er1—N2	106.18 (6)	O9—C22—C23	125.4 (3)
N1—Er1—N2	64.39 (7)	O9—C22—C27	114.9 (3)
O8^i^—Er1—C10	81.95 (6)	C23—C22—C27	119.7 (3)
O11^i^—Er1—C10	102.45 (7)	C30—C29—C24	110.9 (2)
O13—Er1—C10	102.77 (7)	C30—C29—H29A	109.5
O3—Er1—C10	27.15 (7)	C24—C29—H29A	109.5
O4—Er1—C10	26.78 (7)	C30—C29—H29B	109.5
O7—Er1—C10	152.38 (6)	C24—C29—H29B	109.5
O8—Er1—C10	154.90 (6)	H29A—C29—H29B	108.1
N1—Er1—C10	76.13 (7)	C5—C9—C10	114.9 (2)
N2—Er1—C10	95.40 (7)	C5—C9—H9A	108.5
O8^i^—Er1—C20	99.10 (6)	C10—C9—H9A	108.5
O11^i^—Er1—C20	81.86 (7)	C5—C9—H9B	108.5
O13—Er1—C20	73.55 (6)	C10—C9—H9B	108.5
O3—Er1—C20	156.53 (6)	H9A—C9—H9B	107.5
O4—Er1—C20	149.47 (7)	N1—C42—C34	121.8 (3)
O7—Er1—C20	25.58 (6)	N1—C42—C41	118.3 (2)
O8—Er1—C20	26.51 (5)	C34—C42—C41	119.9 (3)
N1—Er1—C20	100.73 (6)	C5—C6—C7	121.5 (3)
N2—Er1—C20	85.77 (6)	C5—C6—H6A	119.3
C10—Er1—C20	175.69 (7)	C7—C6—H6A	119.3
O8^i^—Er1—Er1^i^	38.25 (4)	C22—C23—C24	121.1 (3)
O11^i^—Er1—Er1^i^	69.39 (5)	C22—C23—H23A	119.4
O13—Er1—Er1^i^	69.56 (4)	C24—C23—H23A	119.4
O3—Er1—Er1^i^	123.06 (5)	C38—C39—C40	118.9 (3)
O4—Er1—Er1^i^	109.66 (4)	C38—C39—H39A	120.6
O7—Er1—Er1^i^	86.38 (4)	C40—C39—H39A	120.6
O8—Er1—Er1^i^	34.70 (3)	O10—C27—C22	116.1 (3)
N1—Er1—Er1^i^	146.54 (5)	O10—C27—C26	124.6 (3)
N2—Er1—Er1^i^	134.13 (5)	C22—C27—C26	119.3 (3)
C10—Er1—Er1^i^	120.20 (5)	O1—C2—C3	125.3 (3)
C20—Er1—Er1^i^	60.97 (4)	O1—C2—C7	114.9 (3)
O7—C20—O8	120.1 (2)	C3—C2—C7	119.7 (3)
O7—C20—C19	122.8 (2)	O2—C8—H8A	109.5
O8—C20—C19	117.0 (2)	O2—C8—H8B	109.5
O7—C20—Er1	58.95 (12)	H8A—C8—H8B	109.5
O8—C20—Er1	61.19 (13)	O2—C8—H8C	109.5
C19—C20—Er1	178.08 (16)	H8A—C8—H8C	109.5
C30—O13—Er1	135.18 (14)	H8B—C8—H8C	109.5
C20—O8—Er1^i^	158.37 (18)	C24—C25—C26	120.6 (3)
C20—O8—Er1	92.29 (14)	C24—C25—H25A	119.7
Er1^i^—O8—Er1	107.05 (6)	C26—C25—H25A	119.7
O4—C10—O3	121.0 (2)	C2—C3—C4	120.3 (3)
O4—C10—C9	121.2 (2)	C2—C3—H3A	119.9
O3—C10—C9	117.8 (2)	C4—C3—H3A	119.9
O4—C10—Er1	62.56 (13)	C39—C38—C37	119.6 (3)
O3—C10—Er1	58.45 (13)	C39—C38—H38A	120.2
C9—C10—Er1	175.98 (19)	C37—C38—H38A	120.2
C17—O6—C18	116.0 (2)	C25—C26—C27	120.5 (3)
C20—O7—Er1	95.47 (13)	C25—C26—H26A	119.7
C10—O4—Er1	90.66 (14)	C27—C26—H26A	119.7
C12—O5—C11	117.0 (2)	C32—C33—C34	119.9 (3)
O13—C30—O11	126.2 (2)	C32—C33—H33A	120.1
O13—C30—C29	117.7 (2)	C34—C33—H33A	120.1
O11—C30—C29	116.2 (2)	C35—C36—C37	121.1 (3)
C40—N2—C41	117.6 (2)	C35—C36—H36A	119.4
C40—N2—Er1	124.18 (16)	C37—C36—H36A	119.4
C41—N2—Er1	117.48 (17)	C3—C4—C5	120.5 (3)
C10—O3—Er1	94.40 (16)	C3—C4—H4A	119.7
C14—C19—C20	118.0 (2)	C5—C4—H4A	119.7
C14—C19—H19A	107.8	C36—C35—C34	121.7 (3)
C20—C19—H19A	107.8	C36—C35—H35A	119.1
C14—C19—H19B	107.8	C34—C35—H35A	119.1
C20—C19—H19B	107.8	C33—C34—C42	117.9 (3)
H19A—C19—H19B	107.1	C33—C34—C35	123.6 (3)
C2—O1—C1	117.9 (3)	C42—C34—C35	118.5 (3)
C31—N1—C42	118.5 (2)	O9—C21—H21A	109.5
C31—N1—Er1	121.14 (18)	O9—C21—H21B	109.5
C42—N1—Er1	120.17 (16)	H21A—C21—H21B	109.5
N1—C31—C32	122.7 (3)	O9—C21—H21C	109.5
N1—C31—H31A	118.6	H21A—C21—H21C	109.5
C32—C31—H31A	118.6	H21B—C21—H21C	109.5
N2—C41—C37	122.1 (3)	C33—C32—C31	119.1 (3)
N2—C41—C42	118.3 (2)	C33—C32—H32A	120.4
C37—C41—C42	119.6 (2)	C31—C32—H32A	120.4
O6—C17—C16	124.7 (2)	O5—C11—H11A	109.5
O6—C17—C12	116.2 (2)	O5—C11—H11B	109.5
C16—C17—C12	119.1 (2)	H11A—C11—H11B	109.5
C17—C16—C15	119.7 (2)	O5—C11—H11C	109.5
C17—C16—H16A	120.1	H11A—C11—H11C	109.5
C15—C16—H16A	120.1	H11B—C11—H11C	109.5
C13—C12—O5	125.2 (2)	O1—C1—H1A	109.5
C13—C12—C17	119.8 (2)	O1—C1—H1B	109.5
O5—C12—C17	115.0 (2)	H1A—C1—H1B	109.5
C25—C24—C23	118.8 (3)	O1—C1—H1C	109.5
C25—C24—C29	119.6 (2)	H1A—C1—H1C	109.5
C23—C24—C29	121.5 (3)	H1B—C1—H1C	109.5
C13—C14—C15	117.4 (2)	C30—O11—Er1^i^	137.08 (17)
C13—C14—C19	121.5 (2)		
			
O8^i^—Er1—N1—C31	15.8 (3)	O8^i^—Er1—C10—O3	−104.49 (15)
O11^i^—Er1—N1—C31	166.0 (2)	O11^i^—Er1—C10—O3	−31.16 (15)
O13—Er1—N1—C31	−33.4 (2)	O13—Er1—C10—O3	−177.93 (14)
O3—Er1—N1—C31	98.9 (2)	O4—Er1—C10—O3	−177.6 (2)
O4—Er1—N1—C31	45.5 (2)	O7—Er1—C10—O3	92.9 (2)
O7—Er1—N1—C31	−113.9 (2)	O8—Er1—C10—O3	−104.1 (2)
O8—Er1—N1—C31	−91.4 (2)	N1—Er1—C10—O3	107.35 (16)
N2—Er1—N1—C31	176.0 (2)	N2—Er1—C10—O3	45.44 (15)
C10—Er1—N1—C31	72.9 (2)	Er1^i^—Er1—C10—O3	−104.38 (14)
C20—Er1—N1—C31	−104.0 (2)	C11—O5—C12—C13	13.9 (4)
Er1^i^—Er1—N1—C31	−51.5 (3)	C11—O5—C12—C17	−167.2 (3)
O8^i^—Er1—N1—C42	−169.36 (16)	O8^i^—Er1—O13—C30	22.5 (2)
O11^i^—Er1—N1—C42	−19.2 (2)	O11^i^—Er1—O13—C30	−25.5 (3)
O13—Er1—N1—C42	141.41 (19)	O3—Er1—O13—C30	99.4 (2)
O3—Er1—N1—C42	−86.30 (19)	O4—Er1—O13—C30	100.5 (2)
O4—Er1—N1—C42	−139.6 (2)	O7—Er1—O13—C30	−107.6 (2)
O7—Er1—N1—C42	60.95 (18)	O8—Er1—O13—C30	−53.9 (2)
O8—Er1—N1—C42	83.4 (2)	N1—Er1—O13—C30	173.4 (2)
N2—Er1—N1—C42	−9.14 (17)	N2—Er1—O13—C30	−149.1 (2)
C10—Er1—N1—C42	−112.2 (2)	C10—Er1—O13—C30	100.7 (2)
C20—Er1—N1—C42	70.81 (19)	C20—Er1—O13—C30	−81.7 (2)
Er1^i^—Er1—N1—C42	123.31 (17)	Er1^i^—Er1—O13—C30	−17.1 (2)
O8^i^—Er1—N2—C40	−25.0 (3)	O5—C12—C13—C14	−179.6 (3)
O11^i^—Er1—N2—C40	−6.8 (2)	C17—C12—C13—C14	1.5 (4)
O13—Er1—N2—C40	138.47 (19)	C12—C13—C14—C15	2.8 (4)
O3—Er1—N2—C40	−88.8 (2)	C12—C13—C14—C19	−173.6 (2)
O4—Er1—N2—C40	−128.0 (2)	C13—C14—C15—C16	−4.6 (4)
O7—Er1—N2—C40	93.5 (2)	C19—C14—C15—C16	171.8 (3)
O8—Er1—N2—C40	58.7 (2)	C14—C15—C16—C17	2.1 (5)
N1—Er1—N2—C40	179.9 (2)	C15—C16—C17—O6	−177.9 (3)
C10—Er1—N2—C40	−108.3 (2)	C15—C16—C17—C12	2.4 (4)
C20—Er1—N2—C40	75.9 (2)	C18—O6—C17—C16	16.8 (4)
Er1^i^—Er1—N2—C40	34.4 (2)	C18—O6—C17—C12	−163.5 (2)
O8^i^—Er1—N2—C41	164.83 (15)	O5—C12—C17—C16	176.9 (2)
O11^i^—Er1—N2—C41	−176.94 (18)	C13—C12—C17—C16	−4.1 (4)
O13—Er1—N2—C41	−31.7 (2)	O5—C12—C17—O6	−2.8 (3)
O3—Er1—N2—C41	101.10 (18)	C13—C12—C17—O6	176.2 (2)
O4—Er1—N2—C41	61.84 (18)	C15—C14—C19—C20	127.5 (3)
O7—Er1—N2—C41	−76.61 (17)	C13—C14—C19—C20	−56.2 (4)
O8—Er1—N2—C41	−111.39 (17)	Er1—O7—C20—O8	−1.0 (2)
N1—Er1—N2—C41	9.76 (16)	Er1—O7—C20—C19	179.2 (2)
C10—Er1—N2—C41	81.55 (18)	Er1^i^—O8—C20—O7	−153.0 (3)
C20—Er1—N2—C41	−94.28 (17)	Er1—O8—C20—O7	1.0 (2)
Er1^i^—Er1—N2—C41	−135.71 (15)	Er1^i^—O8—C20—C19	26.8 (6)
C1—O1—C2—C3	4.7 (5)	Er1—O8—C20—C19	−179.2 (2)
C1—O1—C2—C7	−174.7 (3)	Er1^i^—O8—C20—Er1	−154.0 (4)
O8^i^—Er1—O3—C10	73.48 (15)	C14—C19—C20—O7	−8.8 (4)
O11^i^—Er1—O3—C10	149.04 (15)	C14—C19—C20—O8	171.4 (2)
O13—Er1—O3—C10	2.63 (18)	O8^i^—Er1—C20—O7	−169.60 (15)
O4—Er1—O3—C10	1.36 (13)	O11^i^—Er1—C20—O7	116.40 (15)
O7—Er1—O3—C10	−129.64 (15)	O13—Er1—C20—O7	−97.38 (15)
O8—Er1—O3—C10	127.95 (15)	O3—Er1—C20—O7	80.5 (2)
N1—Er1—O3—C10	−68.39 (15)	O4—Er1—C20—O7	−93.27 (18)
N2—Er1—O3—C10	−133.14 (16)	O8—Er1—C20—O7	−179.0 (2)
C20—Er1—O3—C10	−174.74 (15)	N1—Er1—C20—O7	−22.72 (16)
Er1^i^—Er1—O3—C10	92.64 (14)	N2—Er1—C20—O7	40.19 (15)
O1—C2—C3—C4	−178.3 (3)	Er1^i^—Er1—C20—O7	−172.71 (17)
C7—C2—C3—C4	1.1 (5)	O8^i^—Er1—C20—O8	9.44 (17)
O8^i^—Er1—O4—C10	−101.61 (14)	O11^i^—Er1—C20—O8	−64.56 (14)
O11^i^—Er1—O4—C10	−40.67 (16)	O13—Er1—C20—O8	81.66 (14)
O13—Er1—O4—C10	179.62 (14)	O3—Er1—C20—O8	−100.4 (2)
O3—Er1—O4—C10	−1.38 (13)	O4—Er1—C20—O8	85.76 (18)
O7—Er1—O4—C10	130.94 (14)	O7—Er1—C20—O8	179.0 (2)
O8—Er1—O4—C10	−141.74 (13)	N1—Er1—C20—O8	156.31 (14)
N1—Er1—O4—C10	96.99 (15)	N2—Er1—C20—O8	−140.77 (14)
N2—Er1—O4—C10	48.17 (15)	Er1^i^—Er1—C20—O8	6.33 (12)
C20—Er1—O4—C10	175.56 (13)	C21—O9—C22—C23	7.2 (6)
Er1^i^—Er1—O4—C10	−118.54 (13)	C21—O9—C22—C27	−172.6 (4)
C2—C3—C4—C5	0.0 (5)	O9—C22—C23—C24	−179.8 (3)
C3—C4—C5—C6	−1.2 (5)	C27—C22—C23—C24	−0.1 (5)
C3—C4—C5—C9	−179.5 (3)	C22—C23—C24—C25	0.5 (4)
C4—C5—C6—C7	1.5 (4)	C22—C23—C24—C29	−179.7 (3)
C9—C5—C6—C7	179.8 (3)	C23—C24—C25—C26	−0.4 (4)
O8^i^—Er1—O7—C20	12.45 (18)	C29—C24—C25—C26	179.9 (3)
O11^i^—Er1—O7—C20	−62.67 (16)	C24—C25—C26—C27	−0.3 (5)
O13—Er1—O7—C20	76.21 (15)	C28—O10—C27—C26	−0.4 (5)
O3—Er1—O7—C20	−139.21 (15)	C28—O10—C27—C22	178.9 (3)
O4—Er1—O7—C20	124.29 (15)	C25—C26—C27—O10	−180.0 (3)
O8—Er1—O7—C20	0.55 (14)	C25—C26—C27—C22	0.8 (5)
N1—Er1—O7—C20	157.08 (16)	O9—C22—C27—O10	−0.1 (4)
N2—Er1—O7—C20	−135.51 (17)	C23—C22—C27—O10	−179.9 (3)
C10—Er1—O7—C20	171.48 (16)	O9—C22—C27—C26	179.2 (3)
Er1^i^—Er1—O7—C20	6.38 (15)	C23—C22—C27—C26	−0.6 (5)
C8—O2—C7—C6	8.8 (5)	C25—C24—C29—C30	−48.6 (3)
C8—O2—C7—C2	−172.4 (3)	C23—C24—C29—C30	131.7 (3)
C5—C6—C7—O2	178.3 (3)	Er1—O13—C30—O11	23.2 (4)
C5—C6—C7—C2	−0.5 (4)	Er1—O13—C30—C29	−154.82 (18)
C3—C2—C7—O2	−179.7 (3)	Er1^i^—O11—C30—O13	−9.5 (4)
O1—C2—C7—O2	−0.3 (4)	Er1^i^—O11—C30—C29	168.52 (17)
C3—C2—C7—C6	−0.8 (4)	C24—C29—C30—O13	103.7 (3)
O1—C2—C7—C6	178.6 (3)	C24—C29—C30—O11	−74.5 (3)
O8^i^—Er1—O8—C20	−170.25 (18)	C42—N1—C31—C32	−0.7 (4)
O11^i^—Er1—O8—C20	109.53 (15)	Er1—N1—C31—C32	174.2 (2)
O13—Er1—O8—C20	−89.87 (14)	N1—C31—C32—C33	−1.5 (5)
O3—Er1—O8—C20	131.40 (15)	C31—C32—C33—C34	1.4 (5)
O4—Er1—O8—C20	−129.55 (14)	C32—C33—C34—C42	0.7 (5)
O7—Er1—O8—C20	−0.53 (13)	C32—C33—C34—C35	−178.3 (3)
N1—Er1—O8—C20	−28.67 (17)	C33—C34—C35—C36	−177.5 (3)
N2—Er1—O8—C20	41.05 (15)	C42—C34—C35—C36	3.5 (5)
C10—Er1—O8—C20	−170.62 (16)	C34—C35—C36—C37	0.2 (5)
Er1^i^—Er1—O8—C20	−170.25 (18)	C35—C36—C37—C38	177.9 (3)
O8^i^—Er1—O8—Er1^i^	0.0	C35—C36—C37—C41	−3.3 (5)
O11^i^—Er1—O8—Er1^i^	−80.22 (7)	C41—C37—C38—C39	−0.2 (4)
O13—Er1—O8—Er1^i^	80.38 (7)	C36—C37—C38—C39	178.6 (3)
O3—Er1—O8—Er1^i^	−58.36 (14)	C37—C38—C39—C40	−1.0 (4)
O4—Er1—O8—Er1^i^	40.69 (11)	C41—N2—C40—C39	−1.1 (4)
O7—Er1—O8—Er1^i^	169.72 (11)	Er1—N2—C40—C39	−171.2 (2)
N1—Er1—O8—Er1^i^	141.58 (8)	C38—C39—C40—N2	1.7 (5)
N2—Er1—O8—Er1^i^	−148.70 (7)	C40—N2—C41—C37	−0.1 (4)
C10—Er1—O8—Er1^i^	−0.4 (2)	Er1—N2—C41—C37	170.66 (18)
C20—Er1—O8—Er1^i^	170.25 (18)	C40—N2—C41—C42	179.2 (2)
C6—C5—C9—C10	−104.0 (3)	Er1—N2—C41—C42	−10.0 (3)
C4—C5—C9—C10	74.2 (4)	C38—C37—C41—N2	0.8 (4)
Er1—O4—C10—O3	2.4 (2)	C36—C37—C41—N2	−178.1 (3)
Er1—O4—C10—C9	−178.5 (2)	C38—C37—C41—C42	−178.5 (2)
Er1—O3—C10—O4	−2.5 (2)	C36—C37—C41—C42	2.6 (4)
Er1—O3—C10—C9	178.3 (2)	C31—N1—C42—C34	3.0 (4)
C5—C9—C10—O4	−7.7 (4)	Er1—N1—C42—C34	−171.98 (19)
C5—C9—C10—O3	171.4 (3)	C31—N1—C42—C41	−176.9 (2)
O8^i^—Er1—C10—O4	73.06 (14)	Er1—N1—C42—C41	8.1 (3)
O11^i^—Er1—C10—O4	146.40 (13)	C33—C34—C42—N1	−3.0 (4)
O13—Er1—C10—O4	−0.38 (14)	C35—C34—C42—N1	176.1 (3)
O3—Er1—C10—O4	177.6 (2)	C33—C34—C42—C41	176.9 (3)
O7—Er1—C10—O4	−89.5 (2)	C35—C34—C42—C41	−4.0 (4)
O8—Er1—C10—O4	73.4 (2)	N2—C41—C42—N1	1.6 (3)
N1—Er1—C10—O4	−75.10 (14)	C37—C41—C42—N1	−179.1 (2)
N2—Er1—C10—O4	−137.01 (14)	N2—C41—C42—C34	−178.3 (2)
Er1^i^—Er1—C10—O4	73.17 (14)	C37—C41—C42—C34	1.0 (4)

Symmetry codes: (i) −*x*+1, −*y*+1, −*z*+1.

### Hydrogen-bond geometry (Å, °)

**Table:**

*D*—H···*A*	*D*—H	H···*A*	*D*···*A*	*D*—H···*A*
C1—H1A···O6^ii^	0.96	2.54	3.312 (4)	137
C16—H16A···O3^iii^	0.93	2.52	3.421 (3)	163
C18—H18C···O3^iii^	0.96	2.38	3.267 (4)	153
C25—H25A···O4	0.93	2.60	3.515 (3)	169
C28—H28A···O1^iv^	0.96	2.48	3.353 (6)	151
C28—H28A···O2^iv^	0.96	2.44	3.220 (5)	138
C33—H33A···O7^v^	0.93	2.37	3.218 (3)	152
C31—H31A···O4	0.93	2.51	2.953 (3)	109
C40—H40A···O11^i^	0.93	2.36	2.998 (4)	125

Symmetry codes: (ii) *x*, *y*−1, *z*+1; (iii) *x*, *y*+1, *z*; (iv) −*x*+1, −*y*, −*z*+2; (v) −*x*, −*y*+1, −*z*+1; (i) −*x*+1, −*y*+1, −*z*+1.
